# Effects of Sprint versus High-Intensity Aerobic Interval Training on Cross-Country Mountain Biking Performance: A Randomized Controlled Trial

**DOI:** 10.1371/journal.pone.0145298

**Published:** 2016-01-20

**Authors:** Allan Inoue, Franco M. Impellizzeri, Flávio O. Pires, Fernando A. M. S. Pompeu, Andrea C. Deslandes, Tony M. Santos

**Affiliations:** 1 Physical Education Department, Estácio de Sá University, Nova Friburgo, RJ, Brazil; 2 Physical Education Center Admiral Adalberto Nunes, Brazilian Navy, Rio de Janeiro, RJ, Brazil; 3 Performance Laboratory, Recife, PE, Brazil; 4 Exercise Psychophysiology Group, School of Arts, Sciences and Humanities, University of São Paulo, São Paulo, SP, Brazil; 5 Schulthess Clinic, Zurich, Switzerland; 6 School of Physical Education and Sports, Federal University of Rio de Janeiro, Rio de Janeiro, RJ, Brazil; 7 Exercise and Sports Sciences Postgraduate Program, Rio de Janeiro State University, Rio de Janeiro, RJ, Brazil; 8 Neuroscience Laboratory of Exercise, Rio de Janeiro, RJ, Brazil; 9 Physical Education Graduate Program and Physical Therapy Graduate Program, Federal University of Pernambuco, Recife, PE, Brazil; Bern University of Applied Sciences, SWITZERLAND

## Abstract

**Objectives:**

The current study compared the effects of high-intensity aerobic training (HIT) and sprint interval training (SIT) on mountain biking (MTB) race simulation performance and physiological variables, including peak power output (PPO), lactate threshold (LT) and onset of blood lactate accumulation (OBLA).

**Methods:**

Sixteen mountain bikers (mean ± SD: age 32.1 ± 6.4 yr, body mass 69.2 ± 5.3 kg and VO_2max_ 63.4 ± 4.5 mL∙kg^-1^∙min^-1^) completed graded exercise and MTB performance tests before and after six weeks of training. The HIT (7–10 x [4–6 min—highest sustainable intensity / 4–6 min—CR100 10–15]) and SIT (8–12 x [30 s—all-out intensity / 4 min—CR100 10–15]) protocols were included in the participants’ regular training programs three times per week.

**Results:**

Post-training analysis showed no significant differences between training modalities (HIT vs. SIT) in body mass, PPO, LT or OBLA (p = 0.30 to 0.94). The Cohen’s *d* effect size (ES) showed trivial to small effects on group factor (p = 0.00 to 0.56). The interaction between MTB race time and training modality was almost significant (p = 0.08), with a smaller ES in HIT vs. SIT training (ES = -0.43). A time main effect (pre- vs. post-phases) was observed in MTB race performance and in several physiological variables (p = 0.001 to 0.046). Co-variance analysis revealed that the HIT (p = 0.043) group had significantly better MTB race performance measures than the SIT group. Furthermore, magnitude-based inferences showed HIT to be of likely greater benefit (83.5%) with a lower probability of harmful effects (0.8%) compared to SIT.

**Conclusion:**

The results of the current study suggest that six weeks of either HIT or SIT may be effective at increasing MTB race performance; however, HIT may be a preferable strategy.

**Trial Registration:**

ClinicalTrials.gov NCT01944865

## Introduction

Aerobic and anaerobic power and capacity are important factors underlying performance in mountain biking (MTB) races [1,2]. Studies [1,3–5] have shown that cross-country mountain biking (XCO) is a high-intensity intermittent activity in which both aerobic and anaerobic energy systems are highly required [1,2,6]. The importance of both energy systems in MTB performance has been confirmed by the strong relationship between XCO performance and peak power output (PPO), maximal oxygen uptake (VO_2max_), ventilatory and lactate thresholds (LT) and the ability of repeated anaerobic efforts (5 x 30-second Wingate test) [2,3,5,7,8]. Indeed, it has been suggested that elite mountain bikers with high VO_2max_ values should use MTB training strategies to improve their capacities to sustain high-intensity, submaximal aerobic work [3]. Moreover, MTB cyclists are also required to generate supramaximal efforts (>500 W) during specific phases of a competition [9]. Therefore, MTB training programs should include submaximal efforts at onset of blood lactate accumulation (OBLA), PPO and VO_2max_ intensities, as well as supramaximal intensities (>100% VO_2max_) to improve performance [1].

Two training modes broadly used to improve cycling performance are high-intensity aerobic training (HIT) and sprint interval training (SIT). HIT alternates 4–5 min efforts at intensities between 85–95% of peak heart rate (HR_PEAK_) with rest or light exercise periods at 50–75% HR_PEAK_. Conversely, SIT employs short (~30 s), supramaximal (>100% VO_2max_), all-out efforts [10]. Various studies have shown that both modalities are effective at increasing cycling aerobic performance in different individuals [11–14], although different mechanisms have been attributed to these modalities. While HIT may increase aerobic performance by promoting increased skeletal muscle buffering capacity and the ability to sustain high-intensity exercise periods (~90–100% VO_2max_) [15–17], SIT improves muscle oxidative potential [18]. Thus, the strategy that promotes the best results for athletic performance is still a matter of debate.

A direct comparison between SIT and HIT remains to be made, as previous studies investigating these training modes have only evaluated them one at a time. Furthermore, to the best of our knowledge, no studies have examined the effects of HIT and SIT in mountain bikers. From a practical point of view, this type of comparison may help MTB coaches, athletes and practitioners identify the best training mode for MTB performance optimization.

Therefore, the current study compared the effects of HIT and SIT on MTB performance as measured in a simulated MTB race. Additionally, we also verified the effects of these training modes on traditional physiological variables, including PPO, LT and OBLA. We hypothesize that HIT and SIT training modalities may be equally effective at improving PPO, LT, OBLA, and performance in MTB race simulation.

## Materials and Methods

### Ethics statement

All experimental procedures were fully explained to the participants prior to the study, and each provided written consent. This research was approved by the Institutional Ethics Committee of Gama Filho University (#051.2010), and all procedures were performed in accordance with the Declaration of Helsinki. The study was included in the clinical trial registration database of the U.S. National Institutes of Health (ClinicalTrials.gov; NCT01944865). Both the protocol used in this trial and its supporting CONSORT checklist are available as supporting information; see S1 Checklist and S1 Protocol.

### Participants

Sixteen trained, experienced mountain bikers volunteered to participate in the study (Table 1). They consistently trained for six days every week and competed regularly for at least five years when the study was conducted (study period: 27.09.2010–23.12.2010). Based on their PPO and VO_2max_ values, the participants were classified as performance cohort level three (PL3—trained) in accordance with the guidelines published by De Pauw et al. [19]. Data from four of the participants were not included in the final analysis, as one athlete in the SIT group withdrew from the study due to upper respiratory infection, while three in the HIT group missed post-test assessments in the HIT group due to schedule incompatibility.

**Table 1:** Anthropometric, physiological and performance results (mean ± SD) of HIT and SIT groups at baseline.

	HIT (n = 7)		SIT (n = 9)	
Variables	Pre 1	Pre 2	Pre 1	Pre 2
**Age (years)**	34.0 ± 6.7	34.0 ± 6.7	30.6 ± 6.3	30.6 ± 6.3
**Height (cm)**	174.1 ± 2.7	174.1 ± 2.7	176.8 ± 6.7	176.8 ± 6.7
**Body mass (kg)**	68.7 ± 2.9	68.8 ± 2.8	69.6 ± 6.9	69.5 ± 6.8
**VO**_**2max**_ **(L·min**^**-1**^**)**	4.3 ± 0.2	4.5 ± 0.3	4.3 ± 0.5	4.4 ± 0.5
**VO**_**2max**_ **(mL·kg**^**-1**^**·min**^**-1**^**)**	63.1 ± 4.2	65.6 ± 5.6	60.6 ± 4.3	64.0 ± 3.2
**PPO (W)**	299.3 ± 28.5	300.2 ± 23.6	293.6 ± 23.2	296.1 ± 22.9
**PPO (W·kg**^**-1**^**)**	4.4 ± 0.3	4.4 ± 0.3	4.2 ± 0.4	4.3 ± 0.4
**LT (W)**	227.1 ± 24.2	221.7 ± 24.1	215.9 ± 31.0	215.2 ± 25.2
**LT (W·kg**^**-1**^**)**	3.3 ± 0.4	3.2 ± 0.3	3.1 ± 0.4	3.1 ± 0.4
**OBLA (W)**	264.9 ± 31.9	266.3 ± 28.4	258.3 ± 31.3	258.9 ± 32.8
**OBLA (W·kg**^**-1**^**)**	3.9 ± 0.4	3.9 ± 0.4	3.7 ± 0.4	3.7 ± 0.4
**Race sim. (s)**	6074 ± 461	6109 ± 510	6142 ± 436	6144 ± 461

SD—standard deviation; HIT—high-intensity aerobic interval training; SIT—sprint interval training; VO_2max_—maximal oxygen uptake; PPO—peak power output; LT—lactate threshold; OBLA—onset of blood lactate accumulation; Race sim.—race simulation.

### Study design and randomization

A randomized, pre-post parallel group design was used in the present study. The participants were randomly allocated to either the HIT group or the SIT group via a computer-generated random sequence created by a researcher unfamiliar with the participants. To increase outcome reliability and statistical power, the participants performed two baseline tests interspersed by one week, and the average of the two tests was used for analysis. After the baseline tests, the participants completed six weeks of either a SIT or a HIT program and post-test assessments. The tests consisted of a maximal incremental exercise test and an MTB Olympic race simulation; in both, the participants used their own bicycles, which were outfitted with electromagnetically braked ergometer (Computrainer^™^ Lab 3D, RacerMate, Seattle, USA).

### VO_2max_ test

VO_2max_ was determined through a maximal incremental test. After calibration of the ergometer according to manufacturer recommendations (before each test), the participants performed a 10 min warm-up set at 100 W. Thereafter, the test increased by 30 W every 5 min until exhaustion. Exhaustion was determined when the pedal cadence dropped below 70 rpm [3] despite verbal encouragement, which was provided to ensure the attainment of maximal values. The participants were free to choose their preferred cadence within 70 to 90 rpm [20]. Heart rate was continuously monitored using a telemetric system (Polar^®^ RS 800 CX (Polar Electro, Oy, Finland), while rating of perceived exertion (RPE) was collected at the end of each stage using Borg’s CR100 scale [21]. Furthermore, respiratory gas exchange was measured through a gas analyzer (Vacuumed Vista-Mini CPX analyzer, Ventura, California, USA) in tandem with Vista Turbo Fit 5.1 software (Ventura, California, USA). The analyzer was calibrated as recommended by the manufacturer before each test. VO_2max_ was calculated as the highest VO_2_ over 30 s averages during the maximal incremental test. PPO was defined as the highest power achieved during the test in accordance with methods published by Kuipers et al. [22].

### Determination of lactate thresholds

During the final 30 s of each stage, blood samples (25 μL) were collected from the participants’ ear lobes and immediately analyzed to determine lactate concentrations. An electro-enzymatic analyzer (YSI^®^ 1500 Sport, Yellow Springs Instruments, Yellow Springs, OH) was calibrated before each test according to the manufacturer's instructions. LT and OBLA were determined for each participant: LT was defined as the power output that elicited a 1 mmol∙L^-1^ increase in blood lactate concentration over values measured during exercise at 40–60% of VO_2max_, a method described by Hagberg and Coyle [23]. In that study [23], the mean lactate value at exercise intensities between 40–60% VO_2max_ was 1.4 mmol∙L^-1^; thus, the LT was 2.4 mmol∙L^-1^. This method provided an objective, individualized, standardized LT. In addition, OBLA was identified as the power output corresponding to a fixed blood lactate concentration of 4 mmol·L^-1^ [24].

### MTB race simulation test

**Fig 1:**
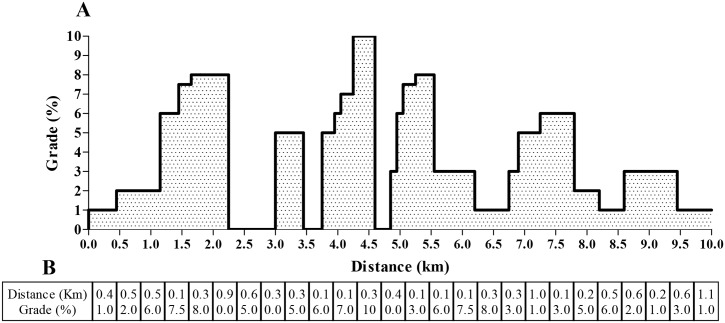
Computer-simulated course showing the (A) overall and (B) specific distance and gradient profile used in this study.

MTB race simulation was conducted in a laboratory environment (temperature between 20–23°C). The participants used their own racing bicycles for the tests, which were outfitted with electromagnetically braked ergometers that were calibrated before each test. Each participant performed a 10 min warm-up at 100 W, thereafter, they completed the MTB test, which was designed to replicate a typical MTB race, including gradient changes through different ascent and descent routes (Fig 1). The MTB race simulation consisted of four 10 km laps with inclination from 0 to 10%. The distance and gradient were set to provide a completion time of ~25 min per lap, for a total of ~100 min per test. Laboratory data (unpublished results) indicated that this MTB simulation race was well correlated with a real XCO competition (*r* = -0.84; p<0.001), with a high intraclass correlation coefficient (ICC = 0.96) and low (1.4%) standard error of measurement. According to Clark et al. [25], the use of simulated cycling time trials with varied gradients offers reliability in terms of time (~1.2%) and power output (~2%), particularly if cyclists perform the trials within a 14-day period. In the present study, we defined changes greater than 1.4% in the time to complete the MTB test as constituting a notable improvement in performance. The participants could watch their race progress on a computer monitor; however, only distance feedback was available. The participants were directed to complete the MTB test as fast as possible according to their self-selected pacing (free gear, cadence and cycling posture (seated or standing)). They consumed water *ad libitum*, and a fan was used to minimize heat stress during the test. HR was continuously measured throughout the test (Polar^®^ RS 800 CX (Polar Electro, Oy, Finland), and the session-RPE [26] was calculated 30 min after the completion of the MTB race [21]. The time to complete the MTB test was recorded and used as a performance measure.

### Other measures

The participants were instructed to fast for three hours and to avoid consuming coffee or stimulants before testing; each test was performed at the same time of day. Body mass, height (Filizola Scale, São Paulo, Brazil), and skinfold thickness (Slim Guide, Rosscraft, Surrey, Canada) were evaluated using standard anthropometric techniques according to International Society for the Advancement of Kinanthropometry (ISAK) guidelines. We estimated body fat percentage using Jackson & Pollock’s equation [27]. Before each session, we obtained the total quality recovery (TQR) for each participant. The TQR scale is based on an athlete’s perception of recovery and is routinely used to monitor recovery, thus reducing the negative effects associated with hard physical training. [28]

### Training program

During the competitive season, the participants trained six times per week, with daily training sessions of 60–180 min (10–15 h wk^-1^). A retrospective analysis of the training during the pre-intervention phase showed that most of the training was performed at low-to-moderate intensity, but at least one high-intensity workout was performed per week. HIT and SIT were included in the participants’ training programs three times per week on non-consecutive days (Tuesday, Thursday and Sunday) for all six weeks of the study (Table 2). In all training sessions, the following steps were included in order: 1) standard warm-up, consisting of 10 min cycling at an intensity corresponding to 10–15 RPE, 10 min cycling at an intensity corresponding to 15–20 RPE, and 20 min cycling at an intensity corresponding to 25–30 RPE; 2) HIT and SIT modalities; 3) cool down period, consisting of 20 min cycling at an intensity corresponding to 10–15 RPE (pedal cadence of 100 rpm). Additionally, the participants used their own bicycles to perform outside training sessions on a road.

**Table 2:** High-intensity interval training program for HIT and SIT groups.

Groups	Weeks	Sessions/ Week	Bouts/ Session	Work duration (min)	Work intensity	Rest duration (min)	Rest intensity (CR100)	Other sessions during the intervention†
HIT*	1	3	8	4	Maximal sustainable	4	10 to 15	60 min—35 RPE
	2	3	10	4		4		60 min—35 RPE
	3	3	3	4		4		40 min—35 RPE
	4	3	7	6		6		60 min—35 RPE
	5	3	8	6		6		60 min—35 RPE
	6	2	2	4		4		30 min—35 RPE
SIT*	1	3	8	0.5	All out	4	10 to 15	60 min—35 RPE
	2	3	10	0.5		4		60 min—35 RPE
	3	3	3	0.5		4		40 min—35 RPE
	4	3	10	0.5		4		60 min—35 RPE
	5	3	12	0.5		4		60 min—35 RPE
	6	2	2	0.5		4		30 min—35 RPE

HIT—high-intensity aerobic interval training; SIT—sprint interval training; CR100—category ratio 0 to 100 scale.

*Every Monday was a day of rest for both groups.

^†^The participants performed the continuous cycling training program reported for each week every Wednesday, Friday and Saturday, as well as a standardized warm-up consisting of 10 min cycling at an intensity corresponding to 10–15 RPE, 10 min cycling at intensity corresponding to 15–20 RPE, and additional 20 min cycling at an intensity corresponding to 25–30 RPE. The cool down consisted of 20 min cycling at an intensity corresponding to 10–15 RPE with a pedal cadence of 100 rpm. On Tuesday of the sixth week, instead of performing interval training (HIT or SIT), the participants in both groups performed 30 minutes of continuous cycling training at an intensity corresponding to 35 RPE.

The HIT and SIT modalities were both performed with an increasing volume base. The HIT modality consisted of 7 to 10 repetitions of 4–6 min at the highest sustainable intensity, followed by the performance of a maximal sprint during the last 30 s of each repetition. Each exercise period was interspersed with recovery periods (4–6 min), which included cycling at intensities corresponding to 10–15 RPE. The training volume progressed by increasing the number of repetitions and the duration of stimulus. The weekly increases included the following: eight repetitions of 4 min in the first week, 10 repetitions of 4 min in the second week, 3 repetitions of 4 min in the third week, 7–8 repetitions of 6 min in the fourth and fifth weeks, and 2 repetitions of 4 min in the sixth week (considered a tapering week).

The SIT modality consisted of 8 to 12 repetitions of a 30 s segment of all-out exercise interspersed with 4 min of recovery, which included cycling at the same intensities as used in the HIT group. The training volume progressed as follows: 8 repetitions of 30 s in the first week, 10 repetitions of 30 s in the second and fourth weeks, 3 repetitions of 30 s in the third week, 12 repetitions of 30 s in the fifth week, and 2 repetitions of 30 s in the sixth week. To avoid possible confounding effects in performance outcomes, no strength or plyometric exercises were included in the training sessions during the study.

### Training quantification

Global training load was quantified based on the training session RPE (session-RPE) [26]. This method quantifies the subjective global training load for each session by multiplying the training duration (min) by the session RPE as measured via Borg’s CR100 scale [21].

### Statistical analysis

Descriptive results were presented as the mean ± standard deviation (SD). After ensuring Gaussian distribution, comparisons of PPO, LT, OBLA and MTB race simulation performance between pre-training (average of two baseline tests) and post-training measurements were performed using two-way analysis of variance (ANOVA) with time (pre- vs. post-training) and training modality (HIT vs. SIT) as fixed factors. Furthermore, analysis of co-variance (ANCOVA) was used to compare post-training outcomes when controlling for pre-training values as a co-factor. Analysis was performed after log-transformation of the dependent variables. We further investigated whether training load should be added as a covariate after performing Pearson’s product moment correlation to assess the relationship between changes in scores for the MTB race simulation and the mean weekly training load. Between-group differences in training load were examined using an independent *t*-test. Significance for inferential analysis was accepted at p<0.05, which was calculated using SPSS version 17 (SPSS, Inc., Chicago, IL, USA).

Combining the actual race performance dataset (n = 16) with the observed effect size (ES) expressed as Cohen’s *d* (ES = -0.43) and an alpha value of 0.05 (two-sided), the power of the test was 0.70. In addition, we estimated the 95% confidence intervals (CI_95%_) corresponding to the pre- and post-training results for both HIT and SIT, classifying the effects as the following: <0.2, trivial; 0.2–0.6, small; 0.6–1.2, moderate; >1.2, large [29] (spreadsheet available at www.cem.org/effect-size-calculator). We further used a magnitude-based inference approach to estimate the chances of higher and lower differences with the following scale: <0.5%, most unlikely or almost certainly not; 0.5–5%, very unlikely; 5–25%, unlikely or probably not; 25–75%, possibly; 75–95%, likely or probably; 95–99.5%, very likely; and >99.5%, most likely or almost certainly [29].

## Results

Analysis of pre- and post-training (Table 3) showed no interactions between training modalities (HIT vs. SIT) in body mass, PPO, LT or OBLA (p from 0.30 to 0.94). Additionally, the ES showed a trivial to small effect on group factor (from 0.00 to 0.56). The interaction between MTB race time and training modality was almost significant (p = 0.08), with a smaller ES in HIT vs. SIT training (ES = -0.43).

**Table 3:** Anthropometric, physiological and performance results (mean ± SD) of HIT and SIT groups before and after six weeks of training.

	HIT (n = 7)		SIT (n = 9)		Significance	
Variables	Pre	Post	Pre	Post	Time	Time x Group
**Body mass (kg)**	68.7 ± 2.7	67.9 ± 2.0	69.6 ± 6.9	68.0 ± 7.0	0.020	0.34
**PPO (W)**	299.8 ± 24.6	323.1 ± 24.0	294.8 ± 22.9	310 ± 22.6	<0.001	0.30
**PPO (W∙kg**^**-1**^**)**	4.4 ± 0.3	4.8 ± 0.4	4.3 ± 0.4	4.6 ± 0.3	<0.001	0.56
**LT (W)**	224.4 ± 23.2	233.7 ± 32.0	215.6 ± 27.3	226.6 ± 29.3	0.087	0.87
**LT (W∙kg**^**-1**^**)**	3.3 ± 0.3	3.4 ± 0.4	3.1 ± 0.4	3.3 ± 0.3	0.046	0.75
**OBLA (W)**	265.6 ± 29.5	275.9 ± 28.7	258.6 ± 31.5	269.4 ± 33.1	0.012	0.94
**OBLA (W∙kg**^**-1**^**)**	3.9 ± 0.4	4.1 ± 0.4	3.7 ± 0.4	4.0 ± 0.4	0.003	0.76
**Race (s)**	6091 ± 478	5785 ± 387	6143 ± 446	5961 ± 417	<0.001	0.08
**TQR pre test (a.u.)**	15.9 ± 0.9	15.4 ± 1.5	15.8 ± 1.1	15.9 ± 1.4	0.610	0.42
**TQR pre sim (a.u.)**	16.2 ± 1.3	16.4 ± 1.4	15.0 ± 1.4	15.8 ± 1.4	0.060	0.26

SD—standard deviation; HIT—high-intensity aerobic interval training; SIT—sprint interval training; PPO—peak power output; LT—lactate threshold; OBLA—onset of blood lactate accumulation; TQR—total quality recovery; pre test—pre graded test; pre sim—pre simulation.

**Fig 2:**
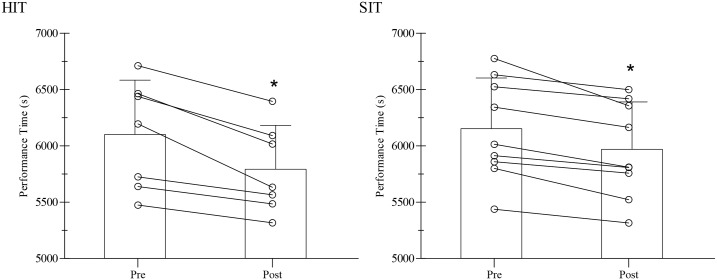
Simulation performance before (PRE) and after (POST) 6 weeks of HIT or SIT. *p<0.001 versus pre-training (main effect for time). Lines denote individual data for 7 subjects in the HIT group and 9 subjects in the SIT group. HIT—high-intensity aerobic interval training; SIT—sprint interval training.

The time main effect (pre- vs. post-phases) indicated that both training modalities were effective at improving both performance (Fig 2) and physiological responses, such as body mass, PPO, LT and OBLA (p from <0.001 to 0.046). The exception was LT expressed as absolute intensity (p = 0.09) (Table 3).

**Fig 3:**
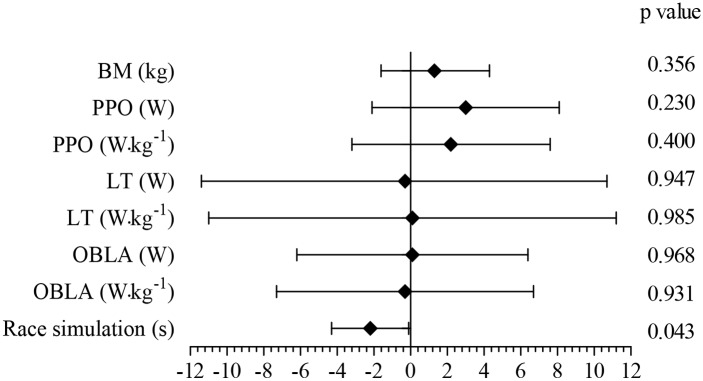
Forrest plot of percentage changes (ANCOVA) with 95% confidence intervals between HIT and SIT. The vertical zero-line represents SIT. BM—body mass; PPO—peak power output; LT—lactate threshold; OBLA—onset of blood lactate accumulation; HIT—high-intensity aerobic interval training; SIT—sprint interval training; Δ %—percentage change.

However, the ANCOVA results revealed that MTB race simulation performance was significantly better in the HIT (p = 0.043) vs. the SIT group (Fig 3). Additionally, a magnitude-based inference approach indicated that the HIT modality was highly beneficial (83.5%) in improving MTB performance compared to the SIT modality; the HIT modality also had a low probability of being harmful (0.8%). The other variables showed differing effects, limiting our conclusions (Table 4).

**Table 4:** Effect size and magnitude-based inference for anthropometric, physiological and performance results of HIT and SIT groups after six weeks of training.

		Magnitude-Based Inference	
Variables	ES Cohen's *d* (CI_95%_)	Beneficial / Negligible / Harmful	Mechanistic inference
**Body mass (kg)**	0.00 (-0.99; 0.99)	0/100/0	Most likely trivial
**PPO (W)**	0.56 (-0.47; 1.54)	75/16.7/8.3	Unclear
**PPO (W∙kg**^**-1**^**)**	0.56 (-0.48; 1.53)	64.3/13.6/22.1	Unclear
**LT (W)**	0.23 (-0.77; 1.21)	50.9/11.2/38	Unclear
**LT (W∙kg**^**-1**^**)**	0.27 (-0.74; 1.25)	53.3/17.7/29	Unclear
**OBLA (W)**	0.20 (-0.80; 1.18)	50/6/44	Unclear
**OBLA (W∙kg**^**-1**^**)**	0.25 (-0.76; 1.22)	52.3/17.7/30	Unclear
**Race (s)**	-0.43 (-1.41; 0.59)	83.5/15.7/0.8	Likely positive for HIT
**TQR pre test (a.u.)**	0.35 (-1.32; 0.67)	63.6/25.7/10.6	Unclear
**TQR pre sim (a.u.)**	0.43 (-0.59; 1.40)	73/21.6/5.4	Unclear

HIT—high-intensity aerobic interval training; SIT—sprint interval training; ES—effect size; CI_95%_—95% confidence interval; PPO—peak power output; LT—lactate threshold; OBLA—onset of blood lactate accumulation; TQR—total quality recovery; pre test—pre graded test; pre sim—pre simulation.

**Fig 4:**
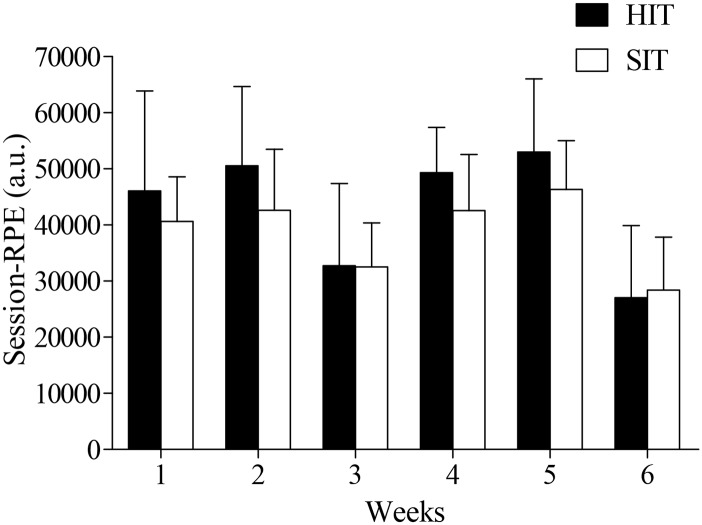
Average weekly training loads of HIT and SIT groups. Calculated using the session rating of perceived exertion (RPE) method, i.e., multiplying the cyclist RPE (using the Category Ratio Scale; CR100) referring to the whole training session by session duration in minutes. HIT—high-intensity aerobic interval training; SIT—sprint interval training; a.u.—arbitrary units.

The weekly training load was 43.110 (12.657) arbitrary units (a.u.) for the HIT group and 38.846 (7.607) a.u. for the SIT group (Fig 4); the difference between them was 4.264 a.u. (CI_95%_ = -7.197 to 15.730; p = 0.436). The session-RPE (CR100) were 83.5 (± 9.6), 90.1 (± 9.7) and 80.2 (± 13.1) for the training sessions with 8 to 10 repetitions of 4 min duration, those with 7 to 8 repetitions for 6 min duration, and those with 8 to 12 repetitions for 30 s duration, respectively. The descriptors of these analyses ranged from very strong to extremely strong. Furthermore, no between-group difference was found in TQR.

## Discussion

To the best of our knowledge, this is the first study showing that trained mountain bikers exhibit enhanced performance in MTB race simulation in addition to improved physiological variables (PPO, LT and OBLA) after participating in training programs incorporating either HIT or SIT. This improvement in performance, which was expressed as the time needed to complete the race simulation, may be due to an increased capacity to maintain high values of power output during MTB race simulation.

Contrary to our initial hypothesis, our ANCOVA results showed that HIT was superior to SIT at enhancing performance in MTB race simulation. A greater improvement in mean power output during MTB race simulation was observed in the HIT group (7.8 ± 3.8%, CI_95%_ = 4.4 to 11.3%) vs. the SIT group (5.0 ± 2.5%, CI_95%_ = 3.1 to 6.9%). Thus, the participants were able to reduce their average completion times of the MTB race simulation by 5.1 ± 2.7 and 3.0 ± 1.8 min in the HIT and SIT groups, respectively, demonstrating that HIT produced better results. These findings provide important information for coaches and athletes about both training modes.

The lack of studies investigating different training interventions in MTB has precluded comparisons of HIT and SIT protocols for mountain biking athletes. However, the improvements in the MTB simulation performance times and PPO values in both the HIT (-4.9 ± 2.4%, CI_95%_ = -7.2 to -2.7%; 7.9 ± 3.5%, CI_95%_ = 4.6 to 11.1%) and SIT groups (-2.9 ± 1.6%, CI_95%_ = -4.1 to -1.7%; 5.3 ± 6.5%, CI_95%_ = 0.3 to 10.3%) are in agreement with previous studies of road cyclists [11,30]. Laursen et al. [11,30] investigated four-week interventions of three different high-intensity interval training programs. After the interventions, the cyclists improved in 40 km time trial performance by 4.4 to 5.8%, together with improvements of 3.0 to 6.2% in PPO and 3.1 to 8.1% in VO_2max_. These results could be related to an improvement in the metabolic glycolytic pathway, as shown by reductions in blood lactate concentrations during the 40-km time trial and by increases in anaerobic capacity as estimated by maximal accumulated oxygen deficit. In addition, oxidative metabolism potentially increased, a change that was supported by the greater VO_2max_ and ventilatory thresholds. Thus, more than one adaptation may be responsible for improving performance following high-intensity interval training in well-trained endurance athletes [30]. However, inferences made based on our results should be undertaken with caution because of the differences that existed between the training programs.

Our results agreed with results from previous studies investigating the impact of different training modalities on cycling performance. Stepto et al. [12] reported that programs of 8 x 4 min at 85% of PPO and 12 x 30 s at 175% of PPO enhanced 40-km time-trial performance. In addition, Seiler et al. [31] showed that a program of 4 x 8 min at 90 ± 2% HR_PEAK_ was more effective at improving cycling performance than training at a lower volume (i.e., 4 x 4 min at 94 ± 2% HR_PEAK_). Similar results were obtained by Sandbakk et al. [32]. In accordance with these results, we observed greater improvement in MTB performance after HIT than SIT, suggesting a relationship between the use of a high percentage of maximal aerobic power and the generation of high, prolonged physical work. Together, these results suggest that the physiological and metabolic benefits observed after training may be influenced by the amount of distance (or time) spent performing at high intensity. Therefore, changes in both intensity and duration during high-intensity sessions may play an important role in endurance adaptations [33].

It has been suggested that exercise intensity is a key factor underlying the activation of peroxisome proliferator-activated receptor gamma coactivator 1 alpha (PGC-1α), a master regulator of mitochondrial biogenesis [34]. It has also been suggested that increases in PGC-1α activation largely arise from increases in muscle recruitment [35] and lactate transporter expression [36]. Weston et al. [17] observed a high correlation between muscle buffer capacity and 40 km time-trial performance (r = -0.82, p<0.05). Furthermore, using different interval training configurations, Westgarth-Taylor et al. [37], Talanian et al. [38] and Burgomaster et al. [39,40] reported enhanced capacity of whole-body and skeletal muscle to oxidize fatty acids, reduced rates of glycogen utilization, and increased total muscle glucose transporter-4 (GLUT-4) protein levels during exercise. In the present investigation, although we did not directly assess muscle metabolism alterations (buffering capacity, whole-body and skeletal muscle capacity for fatty acid oxidation, or markers of mitochondrial biogenesis), we hypothesize that these metabolic alterations led to the improvements in MTB race performance that were observed after the HIT and SIT modalities.

Many coaches and athletes include uphill training in their training routines. High MTB performance during climbing phases requires high aerobic and anaerobic power and capacity, such that mountain bikers must sustain high power output values relative to body mass [3]. In elite cross country MTB races, uphill climbing may represent up to 40% of total race distance [1,41] and 66% of total race time [42]. However, other factors, such as off-road cycling economy, anaerobic power and capacity, and technical ability, might influence off-road cycling performance [1]. Thus, modern MTB races involve multiple factors in addition to climbing ability. Although HIT is likely more beneficial in improving MTB performance in comparison with SIT, both strategies may be effective at increasing MTB race performance and thus both should be considered. While HIT emphasizes power and aerobic capacity for XCO performance (the need to sustain high power output values relative to body mass for prolonged periods at intensities >90% VO_2max_ [43]), SIT focuses on the ability to repeat high-intensity efforts and to produce power at intensities >500 W (such as during steep climbing, at the start of a race and when sprinting to pass slower riders) [9].

As previously reported by others, the present study reported no associations between changes in PPO and performance [16,37]. Based on the results of the accumulated weekly training load, the participants followed the training recommendations (i.e., trial protocol) of the current study, and the reported values of session-RPE (80–90 using the Borg's CR100 scale) showed that the participants trained at the highest sustainable intensity. To the best of our knowledge, this is the first study to calculate session-RPE using Borg's CR100 scale. Future studies should be required to provide session-RPE results.

There were three main limitations in the present study. First, we incorporated 30-s sprints at the end of each bout of exercise during HIT. This strategy was used because we intended 1) to maintain HIT intensity at as high of a level as possible and 2) to improve athlete adherence to the study (interval training routines are preferred in this sport modality). We acknowledge that this strategy may have produced different effects than a more traditional HIT program; in particular, the 30-s sprints may have decreased the magnitudes of differences between the HIT and SIT groups. Nevertheless, the HIT group participants reported the impossibility to perform the 30-s sprints as those in the SIT group due to the total work performed during the previous 4–6 min efforts. Thus, despite this confounding factor (the inclusion of 30-s sprints in the HIT program), we are confident that the groups performed different training programs. In addition, based on the specificities of MTB cross-country racing [1,6], it could be hypothesized that HIT promotes superior physiological adaptations to this sport modality.

Another limitation of note is that no control group was used in the present study, and thus, we could not isolate the additional effects produced by HIT and SIT from those produced by the regular MTB training program. However, this situation is a natural limitation of performing training studies with athletes. Finally, the total work was not equalized between groups, and despite the similar session-RPE values reported for each of the training modes, the HIT group performed ~10% more physical work. Thus, we cannot rule out the possibility that some effects may have been caused by differences in total work. However, the greater amount of physical work performed by the HIT group did not induce lower TQR compared to the SIT group.

## Conclusion

In conclusion, the results of the current study suggest that engagement in 6 weeks of either HIT or SIT may be effective at increasing MTB race performance and other physiological variables. However, based on a magnitude-based inference approach, the HIT modality is likely to be more beneficial (83.5%) at improving MTB performance, with a lower probability of being harmful (0.8%); therefore, HIT may be preferable as a strategy to improve MTB performance. Our results are unclear regarding physiological variables, thus requiring future investigations to better elucidate the differential effects of HIT and SIT in mountain biking athletes.

## Supporting Information

S1 Checklist (DOC)

S1 Protocol (XLS)
